# Breast Cancer in Young Women: Poor Survival Despite Intensive Treatment

**DOI:** 10.1371/journal.pone.0007695

**Published:** 2009-11-11

**Authors:** Hanna Fredholm, Sonja Eaker, Jan Frisell, Lars Holmberg, Irma Fredriksson, Henrik Lindman

**Affiliations:** 1 Department of Molecular Medicine and Surgery, Karolinska Institute, Karolinska University Hospital, Stockholm, Sweden; 2 Regional Oncologic Center, Uppsala University Hospital, Uppsala, Sweden; 3 Department for Surgical Science, Uppsala University Hospital, Uppsala, Sweden; 4 School of Medicine, Division of Cancer Studies, King's College London, London, United Kingdom; 5 Department of Oncology, Radiology and Clinical Immunology, Section of Oncology, Uppsala University Hospital, Uppsala, Sweden; Health Canada, Canada

## Abstract

**Background:**

Breast cancer is uncommon in young women and correlates with a less favourable prognosis; still it is the most frequent cancer in women under 40, accounting for 30–40% of all incident female cancer. The aim of this study was to study prognosis in young women, quantifying how much stage at diagnosis and management on the one hand, and tumour biology on the other; each contribute to the worse prognosis seen in this age group.

**Methodology/Principal Findings:**

In a registry based cohort of women aged 20–69 (n = 22 017) with a primary diagnosis of invasive breast cancer (1992–2005), women aged 20–34 (n = 471), 35–39 (n = 858) and 40–49 (n = 4789) were compared with women aged 50–69 years (n = 15 899). The cumulative 5-year relative survival ratio and the relative excess mortality (RER) were calculated. The cumulative 5-year relative survival ratio was lowest in women aged 20–34. The RER was 2.84 for women aged 20–34 and decreased with increasing age (RER 1.76 and 1.17 for women aged 35–39 and 40–49, respectively). The excess risk was, however, present only in disease stages I and II. For women aged 20–34 with stage I disease RER was 4.63, and 6.70 in the subgroup with tumour size 1–10 mm. The absolute difference in stage I between the youngest and the reference groups amounted to nearly 8%, with a 90% 5-year survival in women aged 20–34. In stages IIa and IIb, the relative excess risk was not as dramatic, but the absolute differences approached 15%. The youngest women with small tumours generally received more aggressive treatment than women in older age groups.

**Conclusions:**

After correction for stage, tumour characteristics and treatment, age remained an independent risk factor for breast cancer death in women <35 years of age. The excess risk for young women was only seen in early stages of disease and was most pronounced in women with small tumours. Young women affected by breast cancer have a high risk of dying compared to their middle-aged counterparts even if diagnosed early and receiving an intense treatment.

## Introduction

Approximately 7% of all breast cancers are diagnosed in women <40 years of age and less than 4% in women below the age of 35 [1], [2], [3]. Although breast cancer is uncommon in young women, it is the most frequent cancer in women <40, accounting for 30–40% of all incident female cancer [4], [5].

Young age at diagnosis influences prognosis negatively [1], [6], [7], [8], [9], [10]. This could partly be explained by young women more often being diagnosed at advanced stages [1], [11] and by unfavourable tumour characteristics more often being present [8], [11], [12], [13], [14], [15]. It has not been quantified how much stage at diagnosis and management on one hand, and tumour biology on the other, each contribute to the poor prognosis.

At the other end of the age spectrum, breast cancer in elderly women is also associated with an inferior prognosis when compared to that of middle-aged women [6], [16]. In a prior study we focused on the elderly and found that less diagnostic activity and less intensive treatment were major explanations for their relatively low survival [17]. We interpreted our findings to be partly due to less rigorous guidelines for the treatment of women aged >70 and were alarmed that unclear guidelines might also contribute to the worse prognosis seen in young women, since guidelines for treating younger women have been less structured than for middle-aged women.

The aim of the present study was to investigate to what degree a worse prognosis in young women can be explained by stage, tumour characteristics and treatment procedures.

## Methods

### 2.1. Participants

Data were collected from the regional breast cancer registers in two of Sweden's six health-care regions (Stockholm/Gotland and Uppsala/Örebro), which currently serve a population of almost 3.9 million inhabitants, thus covering about 43% of the Swedish population. The registers contain prospectively collected data on patient and tumour characteristics, types of treatment and follow-up. The registers are updated continuously by matching with the National Population Register and the mandatory Swedish Cancer Register to ensure a high coverage. The validity of the Swedish Cancer Register has been tested previously and found to be very high for the purpose of breast cancer studies [18], [19].

A cohort of all women aged 20–69 with a primary diagnosis of invasive breast cancer between 1992 and 2005 was followed until the end of 2006. Of the original cohort, 22 017 women were eligible after exclusion of 52 women with less than one month follow-up. Women with bilateral disease at diagnosis were included only once, with the most advanced cancer as the index tumour. For women offered neoadjuvant treatment and those not operated on, staging was based on clinical and preoperative biopsy data. Data on tumour stage were based on the UICC criteria for histopathological TNM stage [20]. The age interval was chosen to allow comparison of young women with middle-aged women, as the latter group is known to have the best survival [10], [21], [22], [23]. The time interval was chosen to represent recent clinical practice and registration procedures, i.e. with homogeneous treatment guidelines and full coverage by regional breast cancer registers.

We chose the cut off at 50 years to define young women and further divided them into three groups (20–34 years, 35–39 years and 40–49 years) in accordance with earlier studies and current treatment recommendations for women <35 years [24]. Women aged 50–69 years served as a comparison group.

All women aged 50–69 years were invited to screening mammography every second year. For women aged 40–49 the routines for screening mammography differed between the two regions, implying that approximately 50% of women aged 40–49 were invited for mammography with an interval of 1.5 years, while the other 50% were not.

### 2.2. Treatment

During the study period treatment for breast cancer was performed according to regional and national guidelines that closely follow international practice. Surgery involved either modified radical mastectomy or breast conserving surgery [25], combined with either a level I and II axillary clearance [26] or a sentinel node biopsy [27].

Radiotherapy to the remaining breast parenchyma up to a total dose of 50–54 Gy (Grey) was recommended as standard treatment after breast conserving surgery. Since 2003 a boost of 10–16 Gy to the tumour bed has been offered to women of 40–45 years. Radiotherapy to the axilla in a dose of 46–54 Gy has been given to women with involved axillary nodes (to all with ≥4 involved nodes and to a majority with 1–3 involved nodes). After mastectomy a total dose of 50–54 Gy has been given to the chest wall if the tumour primarily invaded the pectoral muscle, if extensive multifocality, when tumour size ≥50 mm or smaller if involved axillary nodes.

During the early years of the study period, tamoxifen was not routinely given to premenopausal women [28]. After 1994 tamoxifen was recommended to all women with hormone receptor positive disease but in one of the two regions, only to women with tumours larger than 10 mm. Since 2005 aromatase inhibitors have been recommended postmenopausal women with node positive disease.

Chemotherapy, mainly 5-fluorouracil, epirubicin, cyclophosphamide (FEC), was recommended to all women with node-positive disease or node-negative, hormone receptor negative disease. For node-positive breast cancer docetaxel in sequence with anthracyclines was introduced in 2004.

### 2.3. Ethics

The regional breast cancer data bases used in this study have a general ethic committee admittance from the regional ethics committee at Karolinska Institutet (diary number 03–630) for studies assessing given treatment and outcome based on the retrospective data collected. The data are to be handled and analyzed without possibility to identify individual patients, and no written consents are thus requested. This retrospective study has fully met the stated criteria and has thus been performed under the above mentioned general admission.

### 2.4. Statistical Methods

The studied end-point was 5-year relative survival ratio (RSR). The observation time was defined as the time between the date of diagnosis and death. In the absence of event, the observation time was censored at the date of end of follow up (31 December 2006). The relative survival ratio was calculated by comparing the observed survival of the women in the study population with the expected survival of the general population matched with age, sex, calendar period and county of residency [29]. The general population in this study was represented by all females in the Uppsala/Örebro and Stockholm/Gotland health-care regions stratified by county (8 counties). Data for calculating county-specific life-tables were collected from Statistics Sweden [30]. SAS 9.1 software was used for all statistical analyses.

The Fisher's exact test was used to test the independence between age and tumour and treatment variables (dichotomized into extremes).

To study differences in survival between age groups while adjusting for the confounding factors available in the dataset, i.e., stage (I, IIa, IIb, III, IV, and undefined owing to unknown lymph node status or tumour size) and calendar period of diagnosis (1992–93, 1994–95, 1996–97, 1998–99, 2000–01, 2002–03, 2004–05), we modelled excess mortality using Poisson regression [31]. In addition, information was retrieved on tumour size (1–10 mm, 11–20 mm, 21–50 mm, ≥51 mm, missing), lymph node involvement (positive, negative, missing, 1–3 engaged lymph nodes, ≥4 engaged lymph nodes), tumour grade (I, II, III, missing), hormonal receptor status [estrogen and progesterone (ER/PR); positive, negative, missing], multifocality (yes, missing), surgical treatment [mastectomy, breast conservation (BCS), other operation, none, missing] and prescribed oncological treatment [neoadjuvant chemotherapy, chemotherapy, radiotherapy, endocrine therapy (ET)]. Stratification according to stage was also performed in order to study whether the differences in survival between age groups were consistent across levels of this variable.

We performed a multivariate analysis adjusted by year of diagnosis, stage at diagnosis and oncological treatment (radiotherapy, chemotherapy, ET), stratified on tumour characteristics to evaluate the independent effect of age on survival. Furthermore, we studied differences in survival between the age groups in stage I-IIb while adjusting for the potential determinants by modeling the excess mortality (RER) using Poisson regression. To assess the effect of the different variables separately, as well as in addition to each other, five separate models were made.

## Results

### 3.1. Patient Characteristics

Tumour and treatment characteristics of the 22 017 women included in the study are shown in Table 1. Compared with women aged 50–69 years, women <35 years had larger tumours: (49% were ≥21 mm vs. 27% in the older age group). Their tumours were also more often multifocal (26% vs 20%), high grade (69% vs 24% for grade III) and hormone receptor- negative (33% vs 16%). Fewer women <35 years presented with stage I disease (27% vs 47%). Lymphatic involvement was more common in women <35 years (49% vs 34%) and they displayed a more advanced lymph node status (42% vs 32% having ≥4 involved lymph nodes).

**Table 1:** Distribution of patient, tumour and treatment characteristics.

	Age at diagnosis (years)			
	20–34	35–39	40–49	50–69
	n = 471 (2.1)	n = 858 (3.9)	n = 4789 (21.8)	n = 15899 (72.2)
**Tumour size (mm)**				
1–10	64 (13.6)	129 (15.0)	876 (18.3)	4317 (27.2)
11–20	153 (32.5)	332 (38.7)	1965 (41.0)	6746 (42.4)
21–50	172 (36.5)	288 (33.6)	1441 (30.1)	3698 (23.3)
≥51	35 (7.4)	57 (6.6)	206 (4.3)	470 (3.0)
Missing	47 (10.0)	52 (6.1)	301 (6.3)	668 (4.2)
**Axillary lymph node status**				
Negative	234 (49.7)	410 (47.8)	2624 (54.8)	9661 (60.8)
Positive	205 (43.5)	412 (48.0)	1908 (39.8)	4818 (30.3)
1–3 pos nodes	118 (25.1)	263 (30.7)	1279 (26.7)	3279 (20.6)
≥4 pos nodes	87 (18.5)	149 (17.4)	629 (13.1)	1539 (9.7)
Missing	32 (6.8)	36 (4.2)	257 (5.3)	1420 (8.9)
**Tumour stage**				
I (T1+N0)	126 (26.8)	264 (30.8)	1836 (38.3)	7430 (46.7)
IIa (T1+N1 or T2+N0)	146 (31.0)	262 (30.5)	1395 (29.1)	4179 (26.3)
IIb (T2+N1 or T3+N0)	106 (22.5)	184 (21.5)	826 (17.2)	1877 (11.8)
III (T3+N1 or T4)	55 (11.7)	87 (10.1)	382 (8.0)	678 (4.3)
IV (M1)	15 (3.2)	25 (2.9)	95 (2.0)	271 (1.7)
Undefined	23 (4.9)	36 (4.2)	255 (5.3)	1464 (9.2)
**Tumour grade**				
I	14 (3.0)	42 (4.9)	364 (7.6)	1850 (11.6)
II	38 (8.1)	134 (15.6)	862 (18.0)	3248 (20.4)
III	115 (24.4)	176 (20.5)	575 (12.0)	1610 (10.1)
Missing	304 (64.5)	506 (59.0)	2988 (62.4)	9191 (57.8)
**Hormone receptor status**				
Positive*	219 (46.5)	517 (60.3)	3050 (63.7)	10148 (63.8)
Negative**	156 (33.1)	194 (22.6)	778 (16.2)	2565 (16.1)
Missing	96 (20.4)	147 (17.1)	961 (20.1)	3186 (20.0)
**Multifocal tumour**				
Yes	81 (17.2)	196 (22.8)	788 (16.4)	2006 (12.6)
Missing	156 (33.1)	279 (32.5)	1706 (35.6)	5659 (35.6)
**Surgical treatment**				
Breast Conservation (BCS)	211 (44.8)	413 (48.1)	2658 (55.5)	10151 (63.8)
Mastectomy	224 (47.6)	411 (47.9)	1914 (40.0)	5229 (32.9)
Other operation	3 (0.6)	8 (0.9)	42 (0.9)	81 (0.5)
None	31 (6.6)	26 (3.0)	157 (3.3)	382 (2.4)
Missing	2 (0.4)	0	18 (0.4)	56 (0.4)
**Oncological therapy**				
Neoadjuvant treatment	61 (14.0)	111 (13.5)	387 (8.5)	662 (4.3)
Chemotherapy	307 (65.2)	522 (60.8)	2212 (46.2)	4209 (26.5)
Radiotherapy	335 (71.1)	637 (74.2)	3608 (75.3)	11999 (75.5)
If BCS also radiation	194 (91.9)	387 (93.7)	2486 (93.5)	9382 (92.4)
Endocrine therapy (ET)	192 (40.8)	416 (48.5)	2436 (50.9)	9659 (60.8)
If hormonal positive also ET	151 (69.0)	337 (65.2)	1975 (64.8)	7391 (72.8)

Distribution of patient-, tumour- and treatment characteristics of women aged 20–69 years diagnosed with primary breast cancer of all stages between 1992 and 2005 (22 017 women). Values are numbers (percentages). *ER positive, PR positive or negative, **ER and PR negative**.**

### 3.2. Treatment

When comparing treatment regimes between women <35 years and women of 50–69 years, there were differences as expected from the stage distribution: a larger proportion of women <35 years were treated with mastectomy (48% vs 33%), chemotherapy was more common in women <35 years (65% vs 26%), and use of endocrine therapy was not as common (41% vs 61%) as in the older age group. There were no differences between the age groups in either use of radiotherapy (71% vs 76%) or use of endocrine therapy (in subjects with endocrine responsive tumours 69% vs 73%).

### 3.3. Survival

As of December 31 2006, 3723 (17%) women in our study population had died, of whom 125 were in the age group 20–34 years (26% of all women aged 20–34), 173 aged 35–39 years (20%), 757 aged 40–49 years (16%) and 2668 aged 50–69 years (17%).

The cumulative 5-year relative survival ratio (RSR) was lowest in women <35 years and increased with age. There was a marked distinction in relative excess mortality (RER) in women <35 years compared with older age groups in a model unadjusted for stage (RER 2.84 in comparison with women aged 50–69). In the crude analysis women of 35–49 years also had a worse prognosis compared with the oldest group (Table 2). As expected from the stage distribution in the different age groups, stage at diagnosis was a major explanatory factor for these differences. The adjusted analysis no longer showed a worse survival for the women aged 35–39, but the adjustment did not remove a higher RER for the youngest women.

**Table 2:** Cumulative 5-year relative survival ratio (RSR) and the estimated relative excess risks of mortality (RER) by age.

	Total	5-years survival				Crude^2,4^		Adjusted^3,5^	
	No	Expected	Observed	RSR	95% CI	RER	95% CI	RER	95% CI
Age									
20–34	471	99.8	74.7	74.8	70.1–78.9	2.84	2.31–3.49	1.63	1.32–2.01
35–39	858	99.7	83.8	84.1	81.2–86.6	1.76	1.45–2.14	1.08	0.89–1.32
40–49	4789	99.1	88.3	89.0	88.0–90.0	1.17	1.04–1.31	0.84	0.75–0.94
50–69	15899	96.8	87.8	90.7	90.1–91.2	1.00	(ref.)	1.00	(ref.)

The deviance is a measure of the models goodness-of-fit. Under the hypothesis that the model fits, the deviance should follow a chi-square distribution with the specified degrees of freedom).

2 Likelihood ratio test of the effect of age in the model; df = 3, chi-square = 96.7, p<0.0001.

3 Model adjusted for year (1992–93, 1994–95, 1996–97, 1998–99, 2000–01, 2002–03, 2004–05) and stage (I, IIa, IIb, III, IV, undefined). Likelihood ratio test of the effect of age in the model; df = 3, chi-square = 33.5, p<0.0001.

4 Deviance 26, Residual df 12.

5 Deviance 945, Residual df 760.

Cumulative 5-year relative survival ratio (RSR) and the estimated relative excess risks of mortality (RER) by age with 95% confidence intervals (CI) of women 20–69 years, diagnosed with primary breast cancer of all stages between 1992 and 2005 (22 017 women).

**Figure 1:**
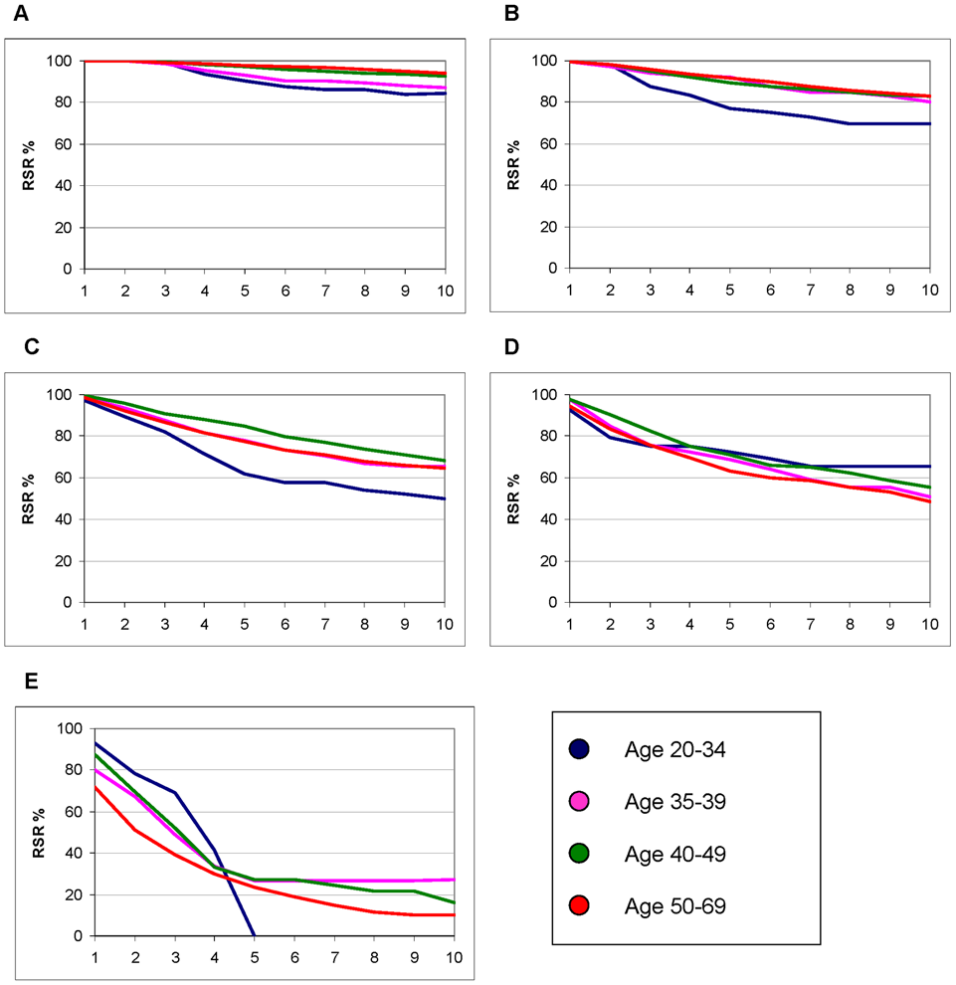
Ten year cumulative survival in relation to expected survival (RSR) according to age and stage of women aged 20–69 years, diagnosed with primary breast cancer between 1992 and 2005 (22 017 women). Size of the groups as in Table 3. A: Stage I, B: Stage IIa, C: Stage IIb, D: Stage III, E: Stage IV.

We further stratified the survival analyses by stage (I, IIa, IIb, III+IV and undefined) to look at stage specific differences (Table 3). Women <35 years with stage I disease had a 4.63-fold excess risk of dying within 5 years. When dividing stage I into tumour sizes 1–10 mm and 11–20 mm, the highest relative excess mortality was seen in the two youngest age groups with the smallest tumours, i.e. 1–10 mm. The absolute difference in stage I between the youngest and the reference groups amounted to nearly 8%, with a 90% 5-year survival in women aged 20–34. In stages IIa and IIb, the relative excess risk was not as dramatic, but the absolute differences approached 15%. In stage III and IV, the RER for the younger women were actually lower than 1.0, but these subgroups are small with limited statistical precision. Figure 1 illustrates the relationship further: the prognosis is generally good in stage I, but there are clear differences between the age groups. Prognosis rapidly becomes worse with stage, with more pronounced absolute differences between the age groups up to and including stage IIb.

**Table 3:** Breast cancer survival by age and stage at diagnosis.

			5-years survival		Crude	
Stage	Age	No.	RSR	95% CI	RER	95% CI
**I**						
	20–34	126	90.1	88.4–94.8	4.63	2.26–9.50
	35–39	264	93.1	88.4–94.8	3.37	1.87–6.09
	40–49	1836	97.1	96.0–98.0	1.40	0.92–2.14
	50–69	7430	97.9	97.3–98.5	1.00	(ref.)
T = 1–10 mm						
	20–34	36	90.9	67.4–97.9	6.70	1.49–30.2
	35–39	78	95.1	84.8–98.6	3.48	0.93–13.0
	40–49	623	99.2	97.6–100.0	0.61	0.18–2.13
	50–69	2881	98.6	97.7–99.5	1.00	(ref.)
T = 11–20 mm						
	20–34	90	90.1	80.0–95.2	3.88	1.73–8.72
	35–39	186	92.3	86.6–95.7	3.11	1.62–6.01
	40–49	1212	96.0	94.4–97.1	1.61	1.03–2.52
	50–69	4542	97.5	96.6–98.2	1.00	(ref.)
**IIA**						
	20–34	146	77.0	68.4–83.5	3.04	2.05–4.50
	35–39	262	92.1	87.6–95.1	1.05	0.64–1.74
	40–49	1395	89.5	87.5–91.2	1.30	1.03–1.63
	50–69	4179	91.9	90.8–92.9	1.00	(ref.)
**IIB**						
	20–34	106	61.7	50.7–71.0	1.78	1.25–2.52
	35–39	184	78.0	70.7–83.8	0.97	0.68–1.37
	40–49	826	84.6	81.7–87.2	0.64	0.51–0.81
	50–69	1877	77.4	75.1–79.5	1.00	(ref.)
**III**						
	20–34	55	72.4	57.5–82.8	0.81	0.47–1.40
	35–39	87	68.6	56.4–78.0	0.86	0.56–1.32
	40–49	382	70.8	65.2–75.7	0.73	0.57–0.93
	50–69	678	63.2	58.8–67.3	1.00	(ref.)
**IV**						
	20–34	15	00.0	-	0.67	0.33–1-37
	35–39	25	26.8	9.8–47.4	0.80	0.48–1.33
	40–49	95	27.1	16.8–38.3	0.74	0.55–1.00
	50–69	271	23.7	17.8–30.1	1.00	(ref.)
**Undefined**						
	20–34	23	77.8	54.4–90.2	2.81	1.13–6-96
	35–39	36	74.3	56.1–85.9	3.04	1.52–6.09
	40–49	255	88.6	83.8–92.1	1.33	0.86–2.06
	50–69	1464	91.3	89.4–92.9	1.00	(ref.)

Cumulative 5-year RSR and the estimated RER and 95% CI by stage at diagnosis of women aged 20–69 years, diagnosed with primary breast cancer of all stages between 1992 and 2005 (22 017 women).

As the excess risk was noted only in young women with the earliest stages of disease (stages I, IIa and IIb), further analyses were restricted to women in these stages. To elucidate the effect of age vs treatment variables on survival, we performed a multivariate analysis stratified on tumour characteristics, correcting for year, stage at diagnosis and oncological treatment (Table 4). We found a significant excess risk of mortality in the women <35 years, compared with the reference group of 50–69 years, in strata with traditionally good prognostic signs such as small tumour size, absence of lymphatic involvement and hormone receptor-positive tumours. The worst relative survival was observed in the youngest women with the smallest tumours. The general pattern was a worse prognosis for women <40 years in nearly all subgroups, but a tendency for women aged 40–49 to do better than those aged 50–69 with the exception of the strata with tumours less than 20 mm.

**Table 4:** The effect of prognostic factors on early breast cancer survival.

	Age at diagnosis (years)						
	20–34		35–39		40–49		50–69
	RER	95% CI	RER	95% CI	RER	95% CI	
**Total**	1.76	1.37–2.26	0.98	0.75–1.27	0.76	0.65–0.89	
Tumour size, 1–10 mm^1^	6.20	2.19–17.53	2.67	0.97–7.33	1.48	0.66–3.29	
Tumour size, 10–20 mm^1^	2.93	1.83–4.69	1.27	0.79–2.04	1.19	0.91–1.54	
Tumour size, 21+ mm^1^	1.41	1.01–1.95	0.77	0.55–1.09	0.58	0.48–0.72	
Lymph nodes, no^2^	2.34	1.49–3.68	1.76	1.13–2.73	0.99	0.75–1.31	
Lymph nodes, yes^2^	1.66	1.22–2.26	0.78	0.56–1.09	0.71	0.59–0.86	
ER+/PR+^3^	2.27	1.47–3.50	1.15	0.77–1.70	0.80	0.62–1.02	
ER−/PR−3	1.32	0.91–1.92	1.00	0.67–1.49	0.92	0.73–1.16	

1 Adjusted for year at diagnosis, lymph node status and oncological treatment.

2 Adjusted for year at diagnosis, tumour size and oncological treatment.

3 Adjusted for year at diagnosis, stage and oncological treatment.

Effect on survival of women aged 20–69 years, diagnosed with primary breast cancer stage I-IIb between 1992 and 2005 (18 631 women), adjusted by year of diagnosis, stage at diagnosis and oncological treatment (radiotherapy, chemotherapy, and endocrine therapy) and stratified on tumour characteristics.

We further addressed the question if the youngest women with small tumours were under-treated, but found, as shown in Table 5, that they generally received more aggressive treatment than women in older age groups.

**Table 5:** Treatments given to women with stage I breast cancer by age and tumour size.

Tumour size	Total	Mastectomy	Chemotherapy	Total no. with BCS	Radiotherapy if BCS	Total no. with hormone positive tumour	Endocrine therapy if hormone positive tumour
	No.	No. (%)	No. (%)	No.	No. (%)	No.	No. (%)
**1–10 mm**							
20–34 years	36	16 (44.4)	8 (22.2)	20	18 (90.0)	22	15 (68.2)
35–39 years	78	22 (28.2)	9 (11.5)	56	49 (87.5)	48	24 (50.0)
40–49 years	623	115 (18.5)	26 (4.2)	503	473 (94.0)	365	161 (44.1)
50–69 years	2881	498 (17.3)	60 (2.1)	2363	2160 (91.4)	1744	951 (54.5)
**11–20 mm**							
20–34 years	90	23 (25.8)	35 (38.9)	66	61 (92.4)	53	39 (73.6)
35–39 years	186	53 (28.7)	70 (37.6)	132	130 (98.5)	123	70 (56.9)
40–49 years	1212	277 (22.9)	212 (17.5)	926	879 (94.5)	859	538 (62.6)
50–69 years	4542	976 (21.5)	422 (9.3)	3542	3351 (94.6)	3296	2344 (71.1)

Proportions of women aged 20–69 years, diagnosed with primary breast cancer stage I between 1992 and 2005 (9656 women), receiving specific treatments, by tumour size and age at diagnosis.

Finally, we undertook a series of multivariate analyses to investigate the combined effect of all potential determinants available in the dataset and to try to elucidate which of the covariates contributed most to the differences (Table 6). In the simplest model, women aged 20–34 years with stage I, IIa and IIb disease had a markedly worse relative survival (RER 3.62) than women aged 50–69 years. Inclusion of year of diagnosis (model 2) changed this estimate little, but introduction of stage (model 3) lowered the RER to 2.35, indicating that stage at diagnosis is an important explanatory variable. After the introduction of all tumour characteristics, the RER dropped to 1.83 (model 4). The final introduction of treatment (model 5) led to a minor shift in the RER estimate to 1.76, thus indicating that tumour characteristics rather that treatment activity is the most important explanatory variable.

**Table 6:** The combined effect of prognostic factors and treatment on breast cancer stage I-IIb.

			Model 1		Model 2		Model 3		Model 4		Model 5	
Age	No.	RSR	RER	95% CI	RER	95% CI	RER	95% CI	RER	95% CI	RER	95% CI
20–34	378	76.7	3.62	2.83–4.63	3.63	2.84–4.66	2.35	1.83–3.01	1.83	1.42–2.35	1.76	1.36–2.28
35–39	710	88.6	1.73	1.34–2.24	1.71	1.32–2.22	1.13	0.87–1.47	1.05	0.81–1.36	1.05	0.80–1.37
40–49	4057	92.0	1.18	1.01–1.37	1.13	0.97–1.32	0.87	0.75–1.01	0.89	0.77–1.04	0.88	0.75–1.03
50–69	13486	93.2	1.00	(ref.)	1.00	(ref.)	1.00	(ref.)	1.00	(ref.)	1.00	(ref.)

Model 1: Crude.

Model 2: Adjusted for year of diagnosis (1992–93, 1994–95, 1996–97, 1998–99, 2001–01, 2002–03, 2004–05).

Model 3: Adjusted for year of diagnosis, tumour stage (tumour size, lymph node status).

Model 4: Adjusted for year of diagnosis, all tumour characteristics (tumour size, lymph node status, tumour grade, hormone receptor status, multifocality).

Model 5: Adjusted for year of diagnosis, all tumour characteristics and treatment (preoperative treatment, type of surgery, radiotherapy, chemotherapy and endocrine therapy).

Estimated RER with 95% CI for women aged 20–69 years, diagnosed with primary breast cancer stage I-IIb between 1992 and 2005 (18 631 women), by age at diagnosis, adjusted for year of diagnosis, tumour stage, tumour characteristics and treatment.

## Discussion

Prognosis in breast cancer has improved dramatically over the past decades, and this study underlines the very good prognosis especially in early stages. As expected from earlier studies women <35 years have a distinctly worse survival than middle-aged women. This study also confirms that women aged 40–49 years have the best survival [6], [16], [22], [23]. Younger women present at a later stage of disease, but that alone does not explain their worse survival since they also have a worse prognosis stage by stage. The distribution of tumour characteristics shown in this study strengthens the assumption that tumour biology is involved. We found no evidence that treatment is less active in the younger women; rather we noticed a higher intensity of treatment corresponding to treatment guidelines. The finding that the age differences in survival are present primarily in stage I and II breast cancer is thought-provoking.

The study population comprises a cohort of consecutive women with primary breast cancer treated according to national guidelines and international practice. The study base is large including a considerable number of young women, conferring a high statistical power for a study in this field. Data were collected from well validated databases in two mainly urban regions of Sweden, and very few women have been lost to follow-up.

The major advantage of using relative survival in this type of analysis is that information on cause of death is not required and that it provides a measure of the excess mortality experienced by patients diagnosed with cancer, irrespective of whether the excess mortality is directly or indirectly attributable to the cancer.

When studying survival by age group in relative terms, one can expect to see large differences as young women with clinically detected tumours with generally more aggressive characteristics stage by stage are being compared with a large group of women with tumours mainly detected by screening mammography with less aggressive characteristics. Still, this reflects that women <35 years, with a normally long life expectancy, will have an absolute risk of dying from their cancer of 25% in such a short follow-up period as 5-year survival. Studies of long-term survival in young women have also shown an increased mortality continuously for up to 40 years after diagnosis [6], [32]. This applies even when breast cancer is diagnosed in a localized stage and in the absence of a second primary breast cancer.

It is remarkable that there are such pronounced age-dependent differences in survival in early breast cancer, which theoretically should be curable. The most striking finding in this study is the high relative excess risk in women <35 years in stage I. Other authors have also found the survival difference by age to be more pronounced in early stages of disease [33], [34]. Kroman et al found the negative effect of young age to be significant only in women with low risk disease who received no adjuvant chemotherapy. It seems reasonable to search for the explanation for these differences in tumour biology.

In our analyses of classical prognostic factors we found the same pattern of more aggressive tumour characteristics in the youngest women as previously published [8], [11], [12], [13], [14], [15]. We lack data on other reported important adverse prognostic factors in the young such as high proliferation index [13], [15], [35], lymphovascular invasion [8], [15], and amplification of the Her-2 gene [14], as well as on preoperative mammography findings with implications for histopathology and prognosis. Tabar et al have reported findings of small tumours, 1–14 mm, showing a mammography pattern with casting-type calcifications being more often present in young women and independently predicting a poor survival [36].

After controlling for different histopathological features most studies have shown young age to remain as a powerful predictor of poor survival [8], [37]. A possible development is that gene expression profiling will be able to differentiate otherwise similar breast cancers at the molecular level to find clues for the explanation of this age effect [38], [39]. Hereditary breast cancer (e.g. BRCA1 and 2 mutations) is more frequent in young women with breast cancer but this has not implied a worse survival in most studies [40]. Other hypotheses to explain the remaining difference in survival between age groups after corrections for tumour characteristics are that the increased risk of local recurrence associated with low age [41], [42], [43] leads to an increased risk of breast cancer death [44] and that young women may differ from older with respect to the treatment they are given and their responsiveness to it, or presumably a combination of both [45], [46], [47].

The young women in the study had been given more intensive treatment than the older women. However, judged against current treatment guidelines, women in all age groups received somewhat suboptimal treatment. Of the women operated with breast conservation, 92–94% were given radiotherapy, while 65–73% of those with hormone receptor-positive tumours received endocrine therapy, which is in line with results from other population-based series [48], [49]. The young women should - according to the 1998 St Gallen guidelines [24] - have received chemotherapy, but only 22% of the women <35 years with stage I disease with tumour size 1–10 mm and 39% with tumour size 11–20 mm did so. The start of our study period several years before the publication of the guidelines might explain the low frequency of chemotherapy. Consequently, there is room for further intensification of the treatment given to all women.

With the results from this study based on a large, well-validated data set, we can conclude that there are two major factors explaining the worse prognosis in young women: late presentation and a smaller, but highly significant component of more aggressive tumour biology. The former underlines the need for a raised awareness of breast cancer in society and among doctors seeing younger patients for breast complaints. The latter triggers several questions: can this aggressiveness be counteracted by even more active treatment with modalities available today, or are new modalities needed? Can we understand the tumour behaviour in the younger women better in order to aid management, e.g. by defining new therapeutic targets? Will such knowledge further improve our understanding of breast cancer biology overall? It would seem that young women are a target group for intensified research of the same importance as e.g. women with triple-negative breast cancer.
